# Sturge-Weber Syndrome: A Case Embedded With All the Features of Spectrum

**DOI:** 10.7759/cureus.36224

**Published:** 2023-03-16

**Authors:** Husam Kivan, Sahar Al Hussein

**Affiliations:** 1 Medicine, Ondokuz Mayis University, Samsun, TUR; 2 General Medicine, Damascus University, Damascus, SYR

**Keywords:** glaucoma, sturge-weber syndrome, seizures, port-wine stain, neurology, birth mark

## Abstract

Sturge-Weber syndrome (SWS) is a rare neurocutaneous vascular disorder characterized by a facial birthmark known as a port-wine stain (PWS), eye abnormalities, and abnormal blood vessels in the brain. It is basically a multisystem disorder that can involve the nervous system, skin, and eyes (phakomatosis). We report a case of a 14-year-old female who presented to the outpatient department with a complaint of upper lip swelling. She had a visible PWS since birth on the left side of her face, which was extending onto the right side of the face as well. She had two episodes of paroxysmal hemiparesis that were four years apart. Moreover, she was diagnosed with epilepsy when she was three years old. She was treated for glaucoma as well when she was nine years old. She was diagnosed with SWS based on her medical history, grossly visible PWS, and neuroimaging findings. Treatment is mostly symptomatic, as no definitive treatment is yet available.

## Introduction

Sturge-Weber syndrome (SWS), also known as encephalotrigeminal angiomatosis, belongs to a group of disorders known as phakomatosis. Phakomatoses, or neurocutaneous disorders, represent a group of disorders in which benign or malignant tumors develop in different parts of the body, mainly the skin, brain, spinal cord, and bones. Port-wine stain (PWS), also known as nevus flammeus, is commonly found in areas innervated by the trigeminal nerve. It extends rarely onto the trunk and upper and lower limbs. SWS is the third most common neurocutaneous disorder after neurofibromatosis and tuberous sclerosis. The most common neurological manifestation of SWS is seizures. SWS can present with variable phenotypes in different individuals [1]. The incidence is one in 50,000-60,000 live births [2]. This syndrome is caused by a somatic mutation in the *GNAQ* gene, on the long arm of chromosome 9 [3]. This mutation is thought to occur sporadically with no identifiable cause [4]. Different symptoms and their severity can vary dramatically from person to person, thus all the cases of SWS are unique in presentation. Both males and females are equally affected by SWS. Moreover, no racial difference in incidence is documented in the literature [4].

SWS can be classified into three subtypes based on symptomatology. Type 1, which accounts for approximately 90% of cases, involves facial and leptomeningeal angiomas. Type 2 is characterized by the presence of a PWS birthmark on the face and may involve the neck and upper chest without intracranial lesions. Both types 1 and 2 may or may not have ocular manifestations, such as glaucoma. Type 3, the rarest subtype, exclusively involves the nervous system and is referred to as the isolated neurological variant. Unlike types 1 and 2, glaucoma is typically not present in type 3.

## Case presentation

A 14-year-old female presented to the outpatient department with a complaint of painless swelling of the upper lip. The swelling was non-pulsatile, non-compressible, and painless. The patient reported that this swelling had recurred in the past but had always resolved on its own. However, during the most recent episode, the swelling persisted for the entire month.

**Figure 1:**
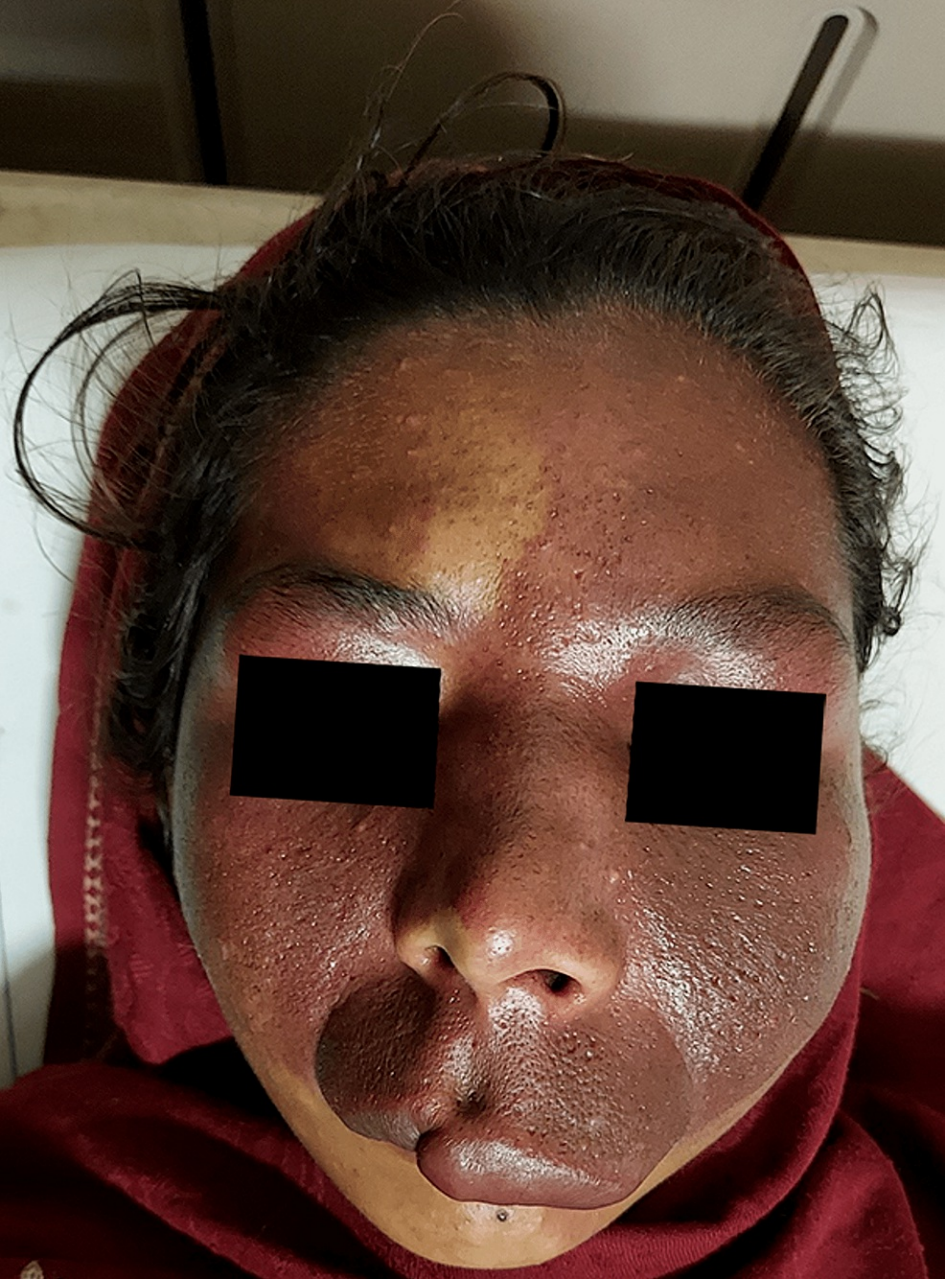
Port-wine stain over the left side of the face also extending onto the right side (along the ophthalmic and maxillary division of the trigeminal nerve).

**Figure 2:**
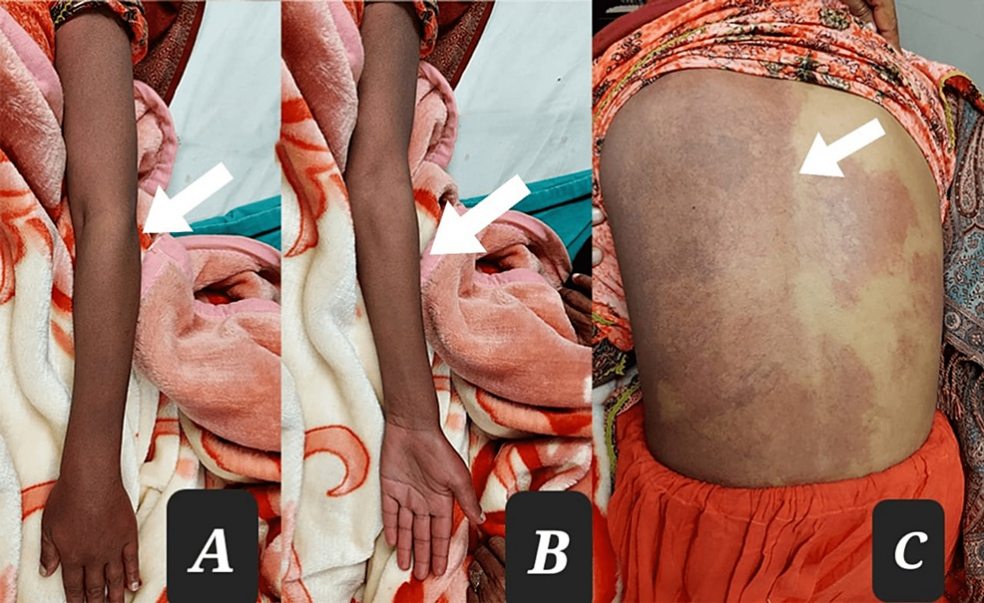
Nevus flammeus involving different parts of the body. Port-wine stain (PWS) involving the ventral aspect of the whole left arm (A), PWS on the dorsal aspect of the whole left arm (B), and PWS on the left side of the whole trunk (C).

The patient had a PWS on the left side of her face that extended onto the right side (Figure 1). This discoloration had been present since birth and anatomically involved the ophthalmic and maxillary divisions of the trigeminal nerve on the left side and the maxillary division only on the right side of the face. The PWS extended over the left side of the trunk, the entire left upper limb, and the left lower limb up to the knee joint, with no involvement of the area below the left knee. A new area of discoloration had also begun to form on the right side of the patient's back (Figure 2). In addition, the patient complained of occasional muscular pain on the left side of her body.

After taking a detailed medical history, the patient disclosed that she had a history of generalized tonic-clonic seizures and was diagnosed with epilepsy at the age of three years. She had been prescribed anticonvulsants to manage her seizures. The patient also reported that a high-grade fever was always associated with her seizures.

The patient had experienced two episodes of right hemiparesis at the ages of five and nine years. Both episodes were completely motor in nature and lasted for a week. She received physical therapy, which helped improve her condition over a month. A neurologist prescribed aspirin for her.

The patient also disclosed that she had been diagnosed with glaucoma in both eyes at the age of nine years due to decreased vision in both eyes. She received treatment for glaucoma at a local hospital and currently reports clear vision with no new episodes since the initial diagnosis.

**Figure 3:**
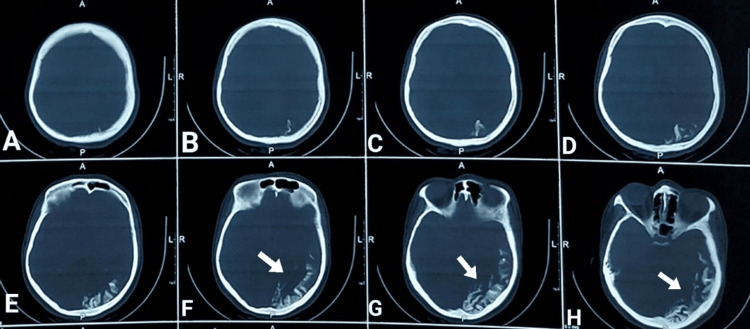
Axial non-contrast CT of the brain showing subcortical calcification in the left occipitoparietal region with a tram-track sign associated with parenchymal volume loss (prominent in images F, G, and H; pointed by white arrows).

On physical examination, the presence of a PWS on the left side of the body, as previously described, was confirmed. A new lesion forming on the right side of the patient's back was also observed. There were no remarkable findings in other systems, including gastrointestinal, cardiovascular, respiratory, and genitourinary. Computed tomography (CT) of the brain revealed subcortical calcification in the left occipitoparietal region with the tram-track sign, which was associated with parenchymal volume loss and ipsilateral choroidal plexus enlargement (Figure 3).

MRI of the brain revealed a decrease in brain volume with mild atrophic changes, which were more pronounced on the left side than on the right. Additionally, there was a dilated lateral ventricle and prominent cortical sulci, ipsilaterally. Other features observed were as follows: (1) coarse cortical tram-track calcification in the parieto-occipital region, as confirmed by a CT scan of the brain; (2) significant leptomeningeal enhancement along the left cerebral hemisphere, particularly in the parieto-occipital region; and (3) thickened areas of the scalp over the left frontal and parietal regions, corresponding to the associated cutaneous lesion.

Finally, based on the patient's medical history, physical examination, and the constellation of imaging findings, a diagnosis of SWS was established.

Regarding the complaint of lip swelling, a lip debulking procedure was planned for the patient under the supervision of a plastic surgery team. Lip reduction surgeries have a better prognosis. After a thorough discussion with the patient and the surgeon's consideration, the Brazilian technique of lip reduction was selected. This procedure focuses on the shape of the lip, creating a more triangular shape at the bottom, resembling a bikini line, and a "bikini top" shape at the upper lip (two cups and a middle strap).

A referral to a dermatologist was made for the extensive PWS, with a recommendation for dry laser photocoagulation. The patient was thoroughly counseled regarding the seizures, episodes of hemiparesis, and glaucoma. The patient was advised to have a continuous follow-up in the surgical outpatient department for the newly formed discolored lesion on the back.

## Discussion

SWS is a group of disorders that can present as multisystem manifestations, mainly neurological, cutaneous, ocular, and oral. Pathologically, all these manifestations arise from vascular malformations, which cause the symptoms mentioned above. Based on the vascular malformation in different systems, the manifestation of SWS can be divided into four distinct parts.

Cutaneous manifestation

PWS is the most common clinical manifestation of SWS [5]. It is also known as nevus flammeus. Commonly, it occurs on the face and is present since birth. It involves the face unilaterally on the right side along the distribution of the ophthalmic division of the trigeminal nerve. However, cases where nevus flammeus involved the face bilaterally have also been reported. Moreover, cases with the distribution of PWS along the other branches of the trigeminal nerve, the maxillary and mandibular branches, have also been documented. In rare instances, small violaceous nodules have been found in the areas of PWS, giving a "cobble-stone" appearance to the cutaneous lesion [6]. These nodules were found to be essentially angiomatous in nature. In the given case, PWS was present since birth along the ophthalmic and maxillary divisions on the left side and along the maxillary division only on the right side.

Neurological manifestation

Leptomeningeal angiomatosis gives rise to neurological signs and symptoms, such as seizures, stroke-like episodes, contralateral hemiparesis, and vascular headache. Seizures are generally of the partial type and occur contralaterally to the cutaneous lesion [7], and are usually a common presenting feature [5]. Intellectual disability is also commonly found in cases of SWS [2], with some studies reporting a mean intelligence quotient (IQ) of 75 [8]. In the given case, the patient had two episodes of right hemiparesis and a known history of epilepsy since the age of three years.

Ocular manifestations

Glaucoma is the most common ocular manifestation. In most cases, the etiology is an anterior chamber angle abnormality [7]. Vascular malformations may involve other ocular structures such as the choroid, retina, episcleral, and conjunctiva. In our patient, ocular involvement was noted, with a history of glaucoma diagnosed at nine years of age in both eyes due to decreased vision. The patient had been treated for glaucoma at a local hospital and reported clear vision with no new episodes following the initial episode.

Other manifestations

In some cases, oral signs and symptoms have been noted. These generally include soft tissue hypertrophy secondary to characteristic vascular malformations. Typically, unilateral vascular hyperplasia of the oral mucosa and gingiva is noted. This feature has been reported in about 60% of the cases in some studies [9]. These changes can range from mild vascular hyperplasia to large masses, which may add to functional disability and aesthetic problems. In our patient, the complaint of lip swelling was addressed by devising a procedure of lip debulking under the supervision of a plastic surgery team.

Based on various manifestations, SWS is classified into three main types by the Roach scale as follows [10]: type 1: angiomas involve the face and the meninges and may be accompanied by glaucoma; type 2: angiomas only involve the face and may be accompanied by glaucoma; type 3: angiomas involve the leptomeninges only, which is an isolated neurological variant of SWS.

Some studies also add a fourth type that is marked by SWS combined with other disorders such as tuberous sclerosis [11].

To diagnose SWS, the different vascular abnormalities in the skin, eyes, and brain are typically demonstrated. Investigations such as CT and MRI of the brain with gadolinium are most useful in addition to skin and eye examination. Brain MRI with gadolinium enhancement is considered the investigation of choice for diagnosing SWS [4]. In our patient's case, the diagnosis of SWS was confirmed through clinical and radiological examinations, including a brain MRI with gadolinium enhancement.

Differential diagnosis in a patient with features of SWS can include Klippel-Trenaunay-Weber syndrome, which is a rare congenital disorder involving malformation of blood vessels in soft tissues, bones, and the lymphatic system. Angio-osteodystrophy syndrome is characterized by a triad of cutaneous malformations, venous varicosities, and bony or soft tissue overgrowth. Rendu-Osler-Weber syndrome is an inherited disorder of the blood vessels that can cause excessive bleeding. Maffucci syndrome is characterized by benign overgrowth of cartilage, skeletal deformities, and cutaneous lesions composed of abnormal blood vessels. However, in the present case, all these possibilities were ruled out on the basis of a thorough history, clinical examination, and investigations.

The treatment of SWS is generally symptomatic and supportive [12]. However, an expert psychological evaluation and counseling of the patient and their parents can be the most important intervention [1]. The management of other features associated with SWS depends on the individual case. For example, eye involvement and leptomeningeal involvement may require specific treatment options. Soft tissue manifestations, such as lip hypertrophy, should be evaluated to determine if they are functional or aesthetic limitations. In the case of our patient, lip hypertrophy was primarily an aesthetic issue, and the surgical procedure described above was chosen as the treatment option.

## Conclusions

This case is unique and important in that it involves all the major signs and symptoms that are described in the literature. It includes left-sided facial PWS along the ophthalmic and maxillary divisions of the trigeminal nerve. Nevus flammeus extends onto the left side of the whole trunk, the left upper limb, and the left lower limb up to the knee. The patient has a positive history of glaucoma, episodes of paroxysmal paralysis, seizures, and soft tissue hypertrophy. As SWS is rare in nature, a case with all the common features in a single patient might prove to be of vital importance in having a better understanding of the pathology and presentation of this syndrome. Multidisciplinary treatment of SWS, including expert psychological evaluation and counseling of patients and their families, is essential for optimal management.
